# Quantitative control of *ASYMMETRIC LEAVES2* expression is critical for leaf axial patterning in *Arabidopsis*

**DOI:** 10.1093/jxb/ert278

**Published:** 2013-09-04

**Authors:** Xiaofan Chen, Hua Wang, Jiqin Li, Hai Huang, Lin Xu

**Affiliations:** National Laboratory of Plant Molecular Genetics, Shanghai Institute of Plant Physiology and Ecology, Shanghai Institutes for Biological Sciences, Chinese Academy of Sciences, 300 Fenglin Road, Shanghai 200032, China

**Keywords:** Arabidopsis, AS2, epigenetic regulation, histone modification, leaf development, polarity formation.

## Abstract

*ASYMMETRIC LEAVES2* (*AS2*) is one of the key genes required for specifying leaf adaxial identity during leaf adaxial–abaxial polarity establishment. Previous data have shown that, in leaf development, *AS2* is directly repressed by an abaxially located transcription factor KANADI1 (KAN1), so that the *AS2* transcripts are restricted only in the adaxial leaf domain. It is shown here that, different from the spatial repression by KAN1, the quantitative repression of *AS2* in the adaxial domain is also critical for ensuring normal leaf pattern formation. By analysing two gain-of-function *as2* mutants, *as2-5D* and *isoginchaku-2D* (*iso-2D*), it is shown that the similar *AS2*-over-expressed phenotypes of these mutants reflect two different kinds of *AS2* misexpression patterns. While *as2-5D* causes disruption of a KAN1-binding site at the *AS2* promoter leading to derepression of *AS2* in the abaxial side but without changing its expression level of a leaf, *iso-2D* results in over-expression of *AS2* but without altering its adaxial expression pattern. In addition, it was found that, in *iso-2D*, levels of histone H3 lysine 27 trimethylation (H3K27me3) and H3K4me3 at the *AS2* locus are significantly reduced and increased, respectively, compared with those in the wild type and *as2-5D*. These results suggest that during leaf patterning, quantitative control of the *AS2* expression level might involve epigenetic regulations.

## Introduction

Leaf primordia emerge from the peripheral zone of the shoot apical meristem (SAM), and start to establish polarity along the adaxial–abaxial, proximodistal, and mediolateral axes immediately after their initiation (Waites and Hudson, 1995; McConnell and Barton, 1998; Bowman *et al.*, 2002). Among them, the establishment of the adaxial–abaxial axis, which is required for subsequent lamina growth and asymmetric development, is of primary importance (Waites and Hudson, 1995; McConnell and Barton, 1998; Bowman *et al.*, 2002), and differentiation of cells along this axis leads to the formation of leaves facilitating photosynthesis (Waites and Hudson, 1995; McConnell and Barton, 1998; Bowman *et al.*, 2002). During the past decade, a number of factors which play important roles in leaf adaxial–abaxial polarity establishment in *Arabidopsis* have been identified (reviewed by Byrne, 2006; Xu *et al.*, 2007; Husbands *et al.*, 2009; Moon and Hake, 2011).

Genes that specify leaf identity in the adaxial domain include the HD-ZIP III family members *PHABULOSA* (*PHB*), *PHAVOLUTA* (*PHV*), and *REVOLUTA* (*REV*) (Talbert *et al.*, 1995; McConnell and Barton, 1998; Zhong and Ye, 1999; McConnell *et al.*, 2001; Otsuga *et al.*, 2001). In addition, two putative transcription factor genes *ASYMMETRIC LEAVES1* (*AS1*) and *AS2* are also critical in promoting cell differentiation in the adaxial leaf domain (Byrne *et al.*, 2000; Iwakawa *et al.*, 2002; Sun *et al.*, 2002; Xu *et al.*, 2002, 2003; Lin *et al.*, 2003). On the other hand, the YABBY (YAB) family genes *FILAMENTOUS FLOWER* (*FIL*) and *YAB3* (Siegfried *et al.*, 1999), the KANADI (KAN) family genes *KAN1* and *KAN2* (Eshed *et al*., 1999, 2001; Kerstetter *et al.*, 2001), and the AUXIN RESPONSE FACTOR (ARF) family genes *ARF3* (also called *ETT*) and *ARF4* (Pekker *et al.*, 2005) specify the abaxial leaf domain. Small RNAs are also involved in leaf adaxial–abaxial patterning. MicroRNA165 and 166 (miR165 and miR166) (Rhoades *et al.*, 2002; Emery *et al.*, 2003; Juarez *et al.*, 2004; Kidner and Martienssen, 2004; Mallory *et al.*, 2004; Williams *et al.*, 2005) and *trans*-acting small interfering RNA tasiR-ARF from the *TAS3* gene (Yoshikawa *et al.*, 2005; Adenot *et al.*, 2006; Fahlgren *et al.*, 2006; Garcia *et al.*, 2006; Xu *et al.*, 2006) post-transcriptionally target the HD-ZIP III and ARF genes transcripts, respectively, during leaf polarity formation. Recent studies also demonstrated that genes that promote cell proliferation in the leaf are also required for adaxial–abaxial polarity formation (Yuan *et al.*, 2010; Horiguchi *et al.*, 2011; Wang *et al.*, 2011; Xu *et al.*, 2012).

The putative transcription factor gene *AS2* encodes a AS2/LOB-domain protein which forms a protein complex with the MYB-domain transcription factor AS1 to specify the adaxial leaf domain (Byrne *et al.*, 2000; Iwakawa *et al.*, 2002; Sun *et al.*, 2002; Xu *et al.*, 2002, 2003; Lin *et al.*, 2003). *AS2* expression is restricted only to the adaxial leaf domain (Iwakawa *et al.*, 2002, 2007) and this *AS2* pattern is caused by an abaxially located transcription factor, KAN1, which binds to the *AS2* promoter in the abaxial leaf domain to repress *AS2* directly (Eshed **et al**., 1999, 2001; Kerstetter *et al.*, 2001; Wu *et al.*, 2008). *as2-5D* is a gain-of-function *as2* mutant that displayed phenotypes resembling transgenic plants that over-express *AS2* (Wu *et al.*, 2008). It was reported that, in the *as2-5D* mutant, a KAN1-binding site at the *AS2* promoter is disrupted, and thus the abaxial expression of *AS2* fails to be normally repressed (Wu *et al.*, 2008).

To understand better the regulation of *AS2* during leaf polarity formation, another gain-of-function *AS2* mutant, *isoginchaku-2D* (*iso-2D*), which is caused by the insertion of a T-DNA vector carrying cauliflower mosaic virus (CaMV) 35S enhancers at the *AS2* locus (Nakazawa *et al.*, 2003), was investigated. It was found that, different from the defective *KAN1* repression in *as2-5D*, *iso-2D* causes *AS2* over-expression and the drastically increased *AS2* transcripts are only accumulated in the leaf adaxial domain. Our data indicate that, similar to the spatial control by *KAN1*, the quantitative control of *AS2* expression is also critical for leaf axial patterning.

## Materials and methods

### Plant materials and growth conditions

*Arabidopsis* mutants *iso-2D* and *as2-5D* are in the Columbia-0 (Col-0) background (Nakazawa *et al.*, 2003; Wu *et al.*, 2008). Plant growth conditions are according to our previous methods (Xu *et al.*, 2003).

### Scanning electron microscopy (SEM), sectioning, *in situ* hybridization, and GUS staining

SEM and thin-section analyses were carried out according to the methods described previously by Xu *et al.* (2003). *In situ* hybridization was performed according to the protocol described previously (Drews *et al.*, 1991; Long and Barton, 1998; Li *et al.*, 2005), and 14-d-old seedlings were used in *in situ* hybridization. The *AS2* probe was made from a full-length cDNA clone in the pBluescript plasmid. The *GUS* and *FIL* probes were made as described previously (Li *et al.*, 2005; Yao *et al.*, 2009). The colour reaction for the detection of the digoxigenin (DIG)-labelled *AS2* probes was carried out for 3 weeks at room temperature because of the low levels of *AS2* transcripts, while that for detection of the DIG-labelled *GUS* and *FIL* probes was carried out for 2 d and 16h, respectively. Primers used in plasmid constructions are listed in Supplementary Table S1 at *JXB* online. GUS staining and plant tissue sectioning were performed as previously described (Xu and Shen, 2008; He *et al.*, 2012).

### Quantitative reverse transcription-polymerase chain reaction (qRT-PCR) and chromatin immunoprecipitation (ChIP)

Total RNA was extracted from the first pair of rosette leaves or shoot apexes of 14-d-old wild-type and mutant plants and cDNA preparation was according to the method described previously by Xu *et al.* (2003). The ChIP experiment was performed as previously described (Xu *et al.*, 2008), using leaves from the 20-d-old wild-type and mutant plants for chromatin extraction. Immunoprecipitation was performed by using the anti-trimethyl-Histone H3 (lys27) antibody (Cat. 07-449, Millipore, USA) or the rabbit polyclonal to Histone H3 (tri methyl K4) antibody (Cat. ab8580, Abcam, UK). Primers used in the PCR reaction are listed in Supplementary Table S1 at *JXB* online.

### Construction of transgenic plants

A DNA fragment of about 4kb containing the *AS2* promoter (–3990 to –1 prior to ATG) was PCR amplified from wild-type Col-0 or *as2-5D* and were subcloned into the *Sal*I and *Bam*HI restriction sites of the pBI101 vector to result in the *AS2*_*pro*_ *:GUS* and *mAS2*_*pro*_ *:GUS* plasmids, respectively. The *35S*_*pro*_ *:AS2*_*pro*_ *:GUS* and *35S*_*pro*_ *:mAS2*_*pro*_ *:GUS* plasmids were constructed by fusing a DNA fragment containing the 35S promoter to the 5′ end at the *Sal*I site of *AS2*_*pro*_ *:GUS* and *mAS2*_*pro*_ *:GUS*, respectively. These plasmids were introduced into wild-type Col-0 by *Agrobacterium*-mediated transformation using the GV3101 strain. Primers used in the molecular cloning are listed in Supplementary Table S1 at *JXB* online.

## Results

### *as2-5D* and *iso-2D* displayed similar leaf developmental defects

**Fig. 1.**
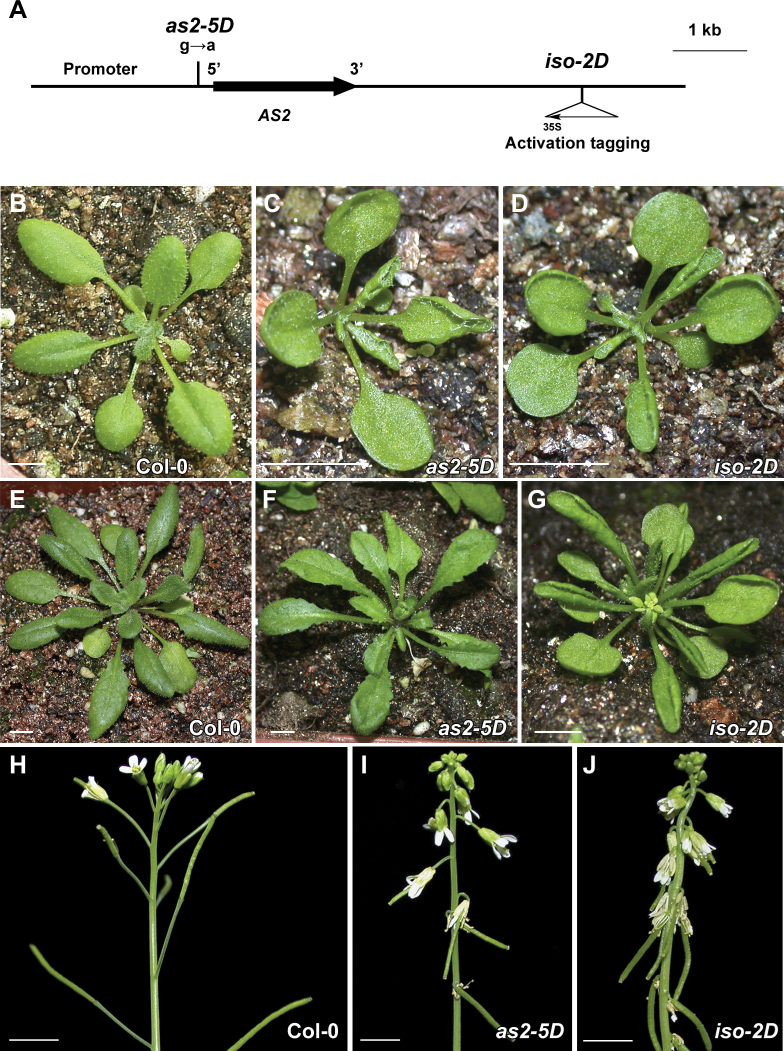
*as2-5D* and *iso-2D* mutants both show *AS2-*over-expression phenotypes. (A) Structure of the *AS2* gene. In *as2-5D*, a nucleotide substitution results in a disrupted KAN1 binding site in the *AS2* promoter. The *iso-2D* mutation is caused by the activation tagging of 35S enhancers. (B–D) Phenotypes of 21-d-old Col-0 (B), *as2-5D* (C), and *iso-2D* (D) seedlings. (E–G) Phenotypes of 30-d-old Col-0 (E), *as2-5D* (F), and *iso-2D* (G) seedlings. (H–J) Inflorescence phenotypes of 45-d-old Col-0 (H), *as2-5D* (I), and *iso-2D* (J) plants. Note that although both *as2-5D* and *iso-2D* mutants show the *AS2-*over-expression phenotypes, the *iso-2D* phenotypes are usually more severe in the later plant developmental stages. Bars=5mm in (B)–(J).

The *iso-2D* mutant carries an activation-tagging T-DNA insertion containing 4×35S enhancers at a position more than 3kb away from the 3΄ end of the *AS2* coding region (Fig. 1A) (Nakazawa *et al.*, 2003). Compared with the wild-type Col-0 (Fig. 1B), the previously characterized *as2-5D* (Wu *et al.*, 2008) and the *iso-2D* mutants showed similar developmental defects at the seedling stage before the 9th leaf was formed (Fig. 1C, D). The phenotypic severity became weaker in *as2-5D* at subsequent plant developmental stages than that in *iso-2D*. For example, both mutant seedlings showed up-curled rosette leaves (Fig. 1C, D) and down-pointing flowers and siliques (Fig. 1I, J). These are the typical *AS2* over-expression phenotypes first observed in the *35S*_*pro*_ *:AS2* transgenic plants (Lin *et al.*, 2003; Xu *et al.*, 2003). However, at the late developmental stages, leaves of *as2-5D* became flat gradually (Fig. 1F), whereas those of *iso-2D* kept severely up-curled (Fig. 1G). In addition, the angles between siliques and stems were larger in *as2-5D* than in *iso-2D*, indicating that this inflorescence phenotype in *as2-5D* is also weaker (Fig. 1I, J).

**Fig. 2.**
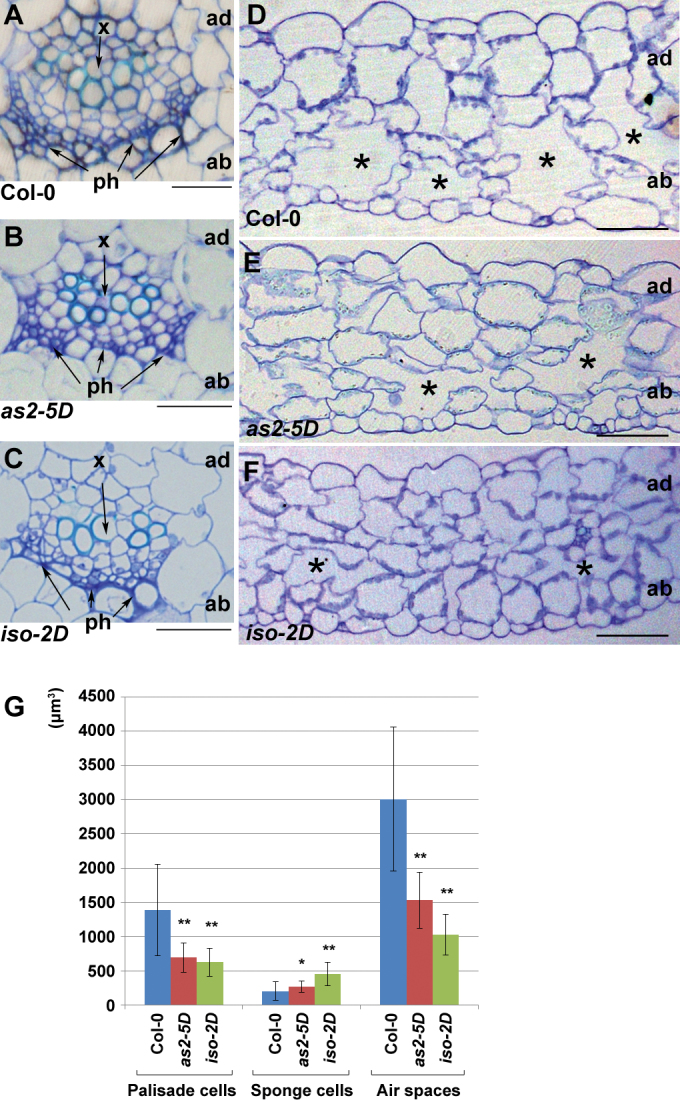
Transverse section analyses of leaf petioles and blades. (A–C) Transverse sections of Col-0 (A), *as2-5D* (B), and *iso-2D* (C) petioles. There were no obvious defects observed in the mutant petioles. (D–F) Transverse sections of Col-0 (D), *as2-5D* (E), and *iso-2D* (F) rosette leaves. Asterisks indicate air spaces. ad and ab, leaf adaxial and abaxial sides, respectively. x, xylem; ph, phloem. Bars = 50 µm in (A)–(F). (G) Quantitative analyses of the cell size and the air space size. The third or fourth rosette leaves from 21-d-old plants were used in sectioning analysis and sections at a position about a quarter of the leaf length from the proximal end of five leaves each were analysed. Cells and air spaces between the first and the second grade branches were scored using the software Image J (http://rsb.info.nih.gov/ij/). Bars show s.d. *, *P <*0.05; **, *P* <0.01.

Transverse sectioning was then performed to analyse vascular and mesophyll patterns of these two mutants. The vascular patterns of both *as2-5D* and *iso-2D* petioles were indistinguishable from that of the wild type, showing that the xylems were on the adaxial pole and phloems on the abaxial pole (Fig. 2A–C). However, the mesophyll patterns in *as2-5D* and *iso-2D* leaves were altered. In the mature wild-type leaves, the adaxially located palisade mesophyll cells appear large and densely packed; whereas the abaxial spongy mesophyll cells are relatively small and are separated by large air spaces (Fig. 2D). In the *as2-5D* leaves, the average size of palisade cells became slightly smaller but that of the spongy mesophyll cells was enlarged with the apparently reduced size of air spaces (Fig. 2E, G). In the *iso-2D* leaves, the reduced size of the adaxial palisade cells and the enlarged size of the abaxial sponge cells became even more pronounced with very small air spaces in the abaxial domain, so that the abaxial leaf domain looked like the adaxial domain (Fig. 2F, G). These mesophyll phenotypes are similar to those described for the adaxialized leaves (Kerstetter *et al.*, 2001; Lin *et al.*, 2003; Grigg *et al.*, 2005).

**Fig. 3.**
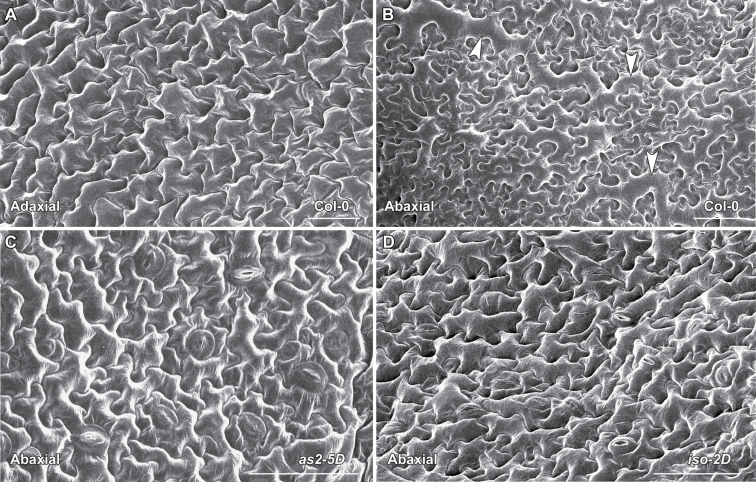
SEM analysis of leaf epidermal cells. (A, B) The wild-type Col-0 leaf epidermal cells on the adaxial (A) or the abaxial (B) side. Arrowheads in (B) indicate the long and large cells which appear only on the leaf abaxial surface. (C, D) The abaxial leaf surface of both *as2-5D* (C) and *iso-2D* (D) contains patches of cells that are similar to the wild-type adaxial epidermal cells, and the long and large abaxially featured cells were not observed in these patches analysed. Bars = 50 µm in (A)–(D).

Leaf epidermal cells of the mutants were then analysed using SEM. The adaxial epidermis of the wild type was composed of uniformly sized cells (Fig. 3A), and the abaxial epidermis was characterized with small pavement cells mixed with long and large cells (Fig. 3B) (McConnell and Barton, 1998). Although the adaxial epidermis appeared normal, patches of the adaxially featured epidermal cells with a uniform cellular size were observed on the abaxial surfaces of both *as2-5D* and *iso-2D* leaves (Fig. 3C, D). All these results from morphological characterization of *as2-5D* and *iso-2D* indicate that both mutants have similar developmental defects while phenotypic abnormalities in *iso-2D* are usually stronger.

### The *as2-5D* and *iso-2D* leaves differ in expression levels of leaf-polarity controlling genes

**Fig. 4.**
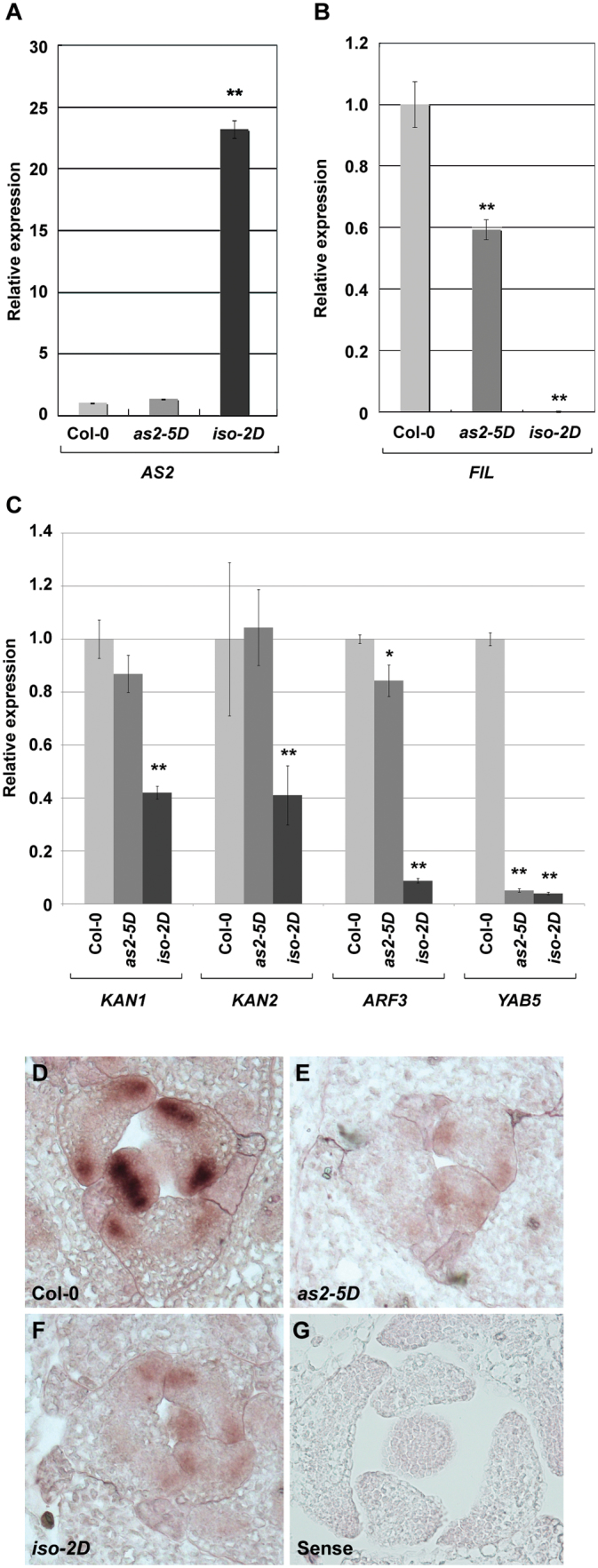
*as2-5D* and *iso-2D* leaves contain different expression levels of polarity genes. (A, B) qRT-PCR to analyse mature leaves for *AS2* (A) and *FIL* (B) expression levels in Col-0, *as2-5D*, and *iso-2D*. (C) qRT-PCR analyses of leaf polarity controlling genes *KAN1*,*KAN2*, *ARF3*, and *YAB5* in Col-0, *as2-5D*, and *iso-2D* mature leaves. The qRT-PCR results were normalized to that produced by the primers at *ACTIN*, and the value of the wild type was arbitrarily fixed at 1.0. Bars show s.e. * and **, significant difference by *t*-test (*, *P <*0.05; **, *P* <0.01). (D–F) *In situ* hybridization to analyse developing leaf primordia for *FIL* expression patterns using an antisense *FIL* probe on transverse sections of Col-0 (D), *as2-5D* (E), and *iso-2D* (F) shoot apices. (G) The *FIL* sense probe control.

To investigate further how these two mutations affect *AS2* expression levels, *AS2* transcript levels in *as2-5D* and *iso-2D* were analysed by qRT-PCR using mature leaves. To our surprise, the total *AS2* transcript level in *as2-5D* leaves was barely changed compared with that in the wild type, whereas that in *iso-2D* leaves was dramatically elevated (Fig. 4A). qRT-PCR was also performed in order to examine the expression levels of a leaf polarity marker gene *FIL* which is normally expressed in the abaxial domain of wild-type leaves (Siegfried *et al.*, 1999). Compared with that in the wild type, the *FIL* expression level was reduced in both *iso-2D* and *as2-5D* at different levels in the mature leaves (Fig. 4B). While the *as2-5D* leaves showed a reduction of about 40%, *FIL* expression in the *iso-2D* leaves was not detected (Fig. 4B). These results are consistent with phenotypic observations that *iso-2D* has more severe defects in mature leaves than *as2-5D* in later seedling stages. In addition to the *FIL* gene, our analysis was extended to several other genes that are known to promote leaf abaxial identity, including *KAN1*, *KAN2*, *ARF3*, and *YAB5*. Our results showed that expression levels of *ARF3* and *YAB5* were reduced in the *as2-5D* and *iso-2D* leaves to different extents and expression levels of *KAN1* and *KAN2* were reduced only in the *iso-2D* leaves (Fig. 4C).

An *in situ* hybridization experiment was also performed to analyse *FIL* transcripts in the leaf primordia of the two mutants. Our data showed that the *FIL* transcript level appeared markedly decreased in leaf primordia of both *as2-5D* and *iso-2D* (Fig. 4E, F) compared with that in the wild type (Fig. 4D), and no hybridization signals were detected in the sense control (Fig. 4G). These results provide a molecular basis for these two mutants to produce the adaxialized leaves.

### *AS2* is up-regulated dramatically only in the adaxial leaf domain in *iso-2D*

**Fig. 5.**
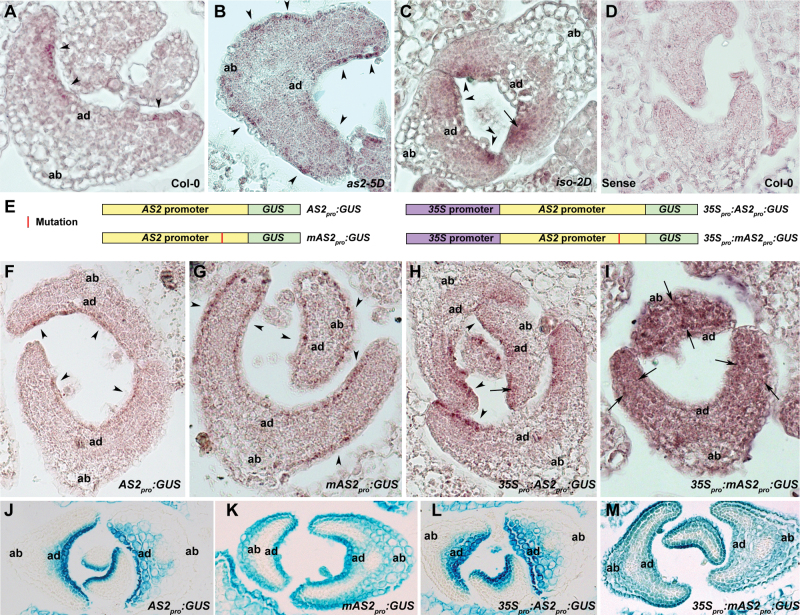
Analyses of the *AS2* expression patterns. (A–C) *In situ* hybridization using an antisense *AS2* probe on transverse sections of leaf primordia to show *AS2* expression patterns in wild-type Col-0 (A), *as2-5D* (B), and *iso-2D* (C). (D) The sense probe control. (E) Diagrams of structures of *AS2*_*pro*_ *:GUS*, *mAS2*_*pro*_ *:GUS*, *35S*_*pro*_ *:AS2*_*pro*_ *:GUS*, and *35S*_*pro*_ *:mAS2*_*pro*_ *:GUS* constructs. (F–I) *In situ* hybridization using an antisense *GUS* probe on transverse sections of leaf primordia to show *GUS* transcript distributions in *AS2*_*pro*_ *:GUS*/Col-0 (F), *mAS2*_*pro*_ *:GUS*/Col-0 (G), *35S*_*pro*_ *:AS2*_*pro*_ *:GUS*/Col-0 (H), and *35S*_*pro*_ *:mAS2*_*pro*_ *:GUS*/Col-0 (I) transgenic plants. Note that five independent transgenic lines for each construct were analysed and the results were consistent, and shown are the results from one of the five lines analysed. (J–M) GUS staining to analyse *AS2* expression. Transverse sections of leaf primordia after GUS staining show GUS distributions in *AS2*_*pro*_ *:GUS*/Col-0 (J), *mAS2*_*pro*_ *:GUS*/Col-0 (K), *35S*_*pro*_ *:AS2*_*pro*_ *:GUS*/Col-0 (L), and *35S*_*pro*_ *:mAS2*_*pro*_ *:GUS*/Col-0 (M) transgenic plants. ad and ab, leaf adaxial and abaxial sides, respectively. Arrowheads and arrows indicate the *AS2* transcripts in the L1 and L2 layers, respectively.

To investigate the mechanism by which *iso-2D* affects leaf polarity formation, the distribution patterns of the *AS2* transcripts were examined at the early leaf developmental stages by *in situ* hybridization. In wild-type leaf primordia, *AS2* transcripts were mainly detected in the L1 layer cells of the adaxial side, showing relatively low hybridization signals that were discontinuously distributed (Fig. 5A). This *AS2* expression pattern is similar to that reported previously (Iwakawa *et al.*, 2007). However, although the *AS2* transcripts in the *as2-5D* leaves were also present mainly in the L1 layer cells, both adaxial and abaxial L1 layer cells contained the *AS2* signals with an intensity similar to that in the wild-type leaves (Fig. 5B). The *AS2* expression pattern in the *as2-5D* mutant is consistent with that using GUS staining of the *AS2-5D*_*pro*_ *:GUS* transgenic plants (Wu *et al.*, 2008). Different from the *AS2* distribution in *as2-5D* leaves, *AS2* was strongly expressed only in the adaxial leaf domain of *iso-2D*, with the strongest hybridization signals in the outermost layers (Fig. 5C). An obvious difference of the hybridization signals between the two mutants is in the L1 layer cells of the abaxial leaf side. Compared with *as2-5D* (Fig. 5B), the abaxial L1 layer cells of *iso-2D* leaf primordia lacked a hybridization signals (Fig. 5C). As a control, the sense *AS2* probe detected no hybridization signals (Fig. 5D).

The *AS2* expression signals detected by *in situ* hybridization were relatively weak. To confirm that these are the true hybridization signals of *AS2* transcripts, three different *GUS* fusions were also constructed as it was expected that the *GUS* transcripts are more stable than those of *AS2* and thus may be easy to detect. These three fusions included: (i) the *GUS* coding region is driven by the *AS2* promoter (*AS2*_*pro*_ *:GUS*); (ii) the *GUS* coding region is driven by the mutated *AS2* promoter as that in the *as2-5D* mutant (*mAS2*_*pro*_ *:GUS*); and (iii) a 35S promoter is fused to the 5΄ end of *AS2*_*pro*_ *:GUS* (*35S*_*pro*_ *: AS2*_*pro*_ *:GUS*) (Fig. 5E). Hence, the *in situ* hybridization signals detected by the *GUS* probe in transgenic lines carrying these three fusions represent *AS2* expression in the wild-type, *as2-5D*, and *iso-2D* leaves, respectively. Our data showed that the distribution patterns of *GUS* signals were fully consistent with that of the endogenous *AS2* transcripts detected by *in situ* hybridization (Fig. 5F–H). For instance, GUS signals were present in the adaxial L1 layer of the *AS2*_*pro*_ *:GUS/*Col-0 (Fig. 5F), the entire L1 layer of the *mAS2*_*pro*_ *:GUS/*Col-0 (Fig. 5G), and more strongly in the adaxial L1 layer of the *35S*_*pro*_ *:AS2*_*pro*_ *:GUS*/Col-0 (Fig. 5H) leaves. *GUS* distributions were also analysed by GUS staining (Fig. 5J–L) and it was found that the GUS distribution pattern between *in situ* hybridization and staining analyses is consistent. The only difference between the two methods is that the GUS signals by GUS staining are not as concentrated as those by *in situ* hybridization.

To test the respective effects of *as2-5D* and *iso-2D* mutations on the *AS2* regulation, the *35S*_*pro*_ *:mAS2*_*pro*_ *:GUS*/Col-0 transgenic plants were constructed with a genetic background equivalent to that of the *as2-5D iso-2D* double mutant (Fig. 5E). *In situ* hybridization and GUS staining analyses both showed that the GUS signals were present in the entire leaf primordium (Fig. 5I, M), indicating that the mechanisms controlling *AS2* expression in *as2-5D* and *iso-2D* are different.

### Histone methylation patterns are changed at the *AS2* locus in the *iso-2D* mutant

**Fig. 6.**
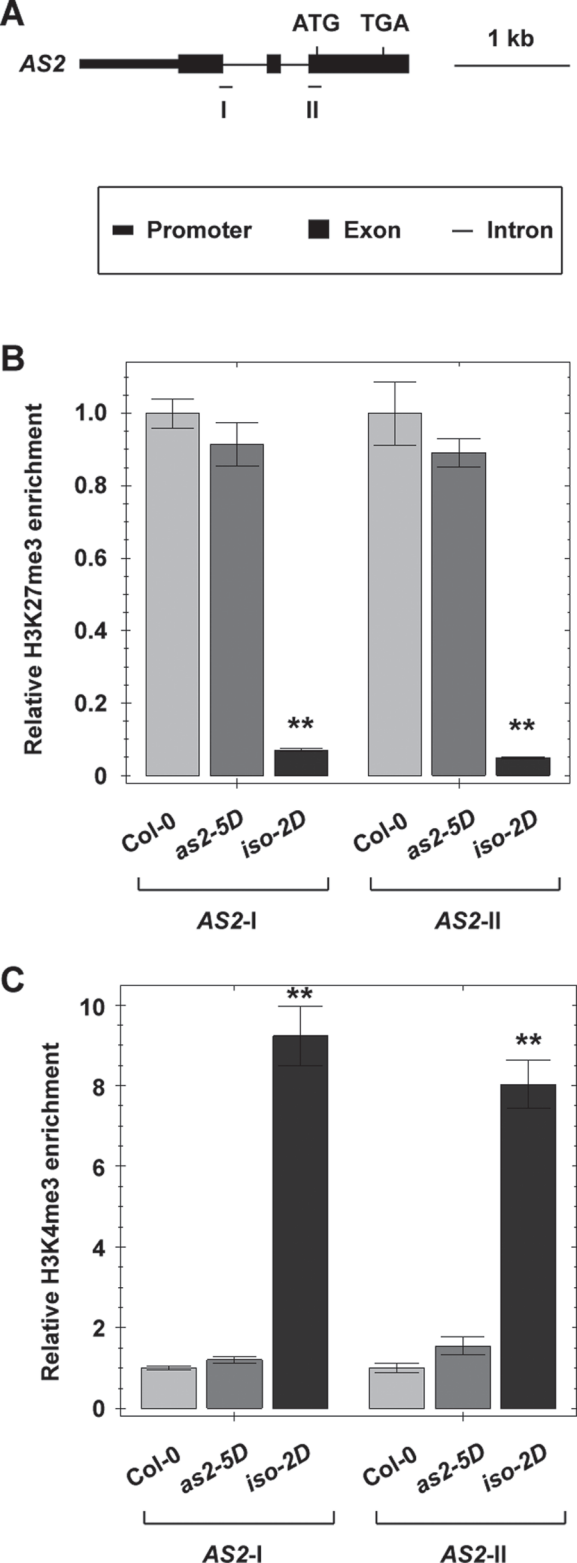
*iso-2D* leaves carry altered levels of histone modification markers H3K27me3 and H3K4me3 at the *AS2* locus. (A) Diagram of the *AS2* gene with primer positions (I and II) in ChIP analysis. (B, C) Compared with those in Col-0 and *as2-5D*, the H3K27me3 level in the *iso-2D* mutant was dramatically reduced (B) whereas the H3K4me3 level was elevated (C) at the *AS2* locus. The ChIP results were normalized to those produced by the primers at *PI* (B) and *ACTIN* (C). Values of the wild type were arbitrarily fixed at 1.0. Bars show s.e. **, significant difference by *t*-test (*P* <0.01).

Histone methylations, especially the histone H3 lysine 27 trimethylation (H3K27me3) and H3K4me3, are usually thought to be important in regulating gene expression in the euchromatin region (Liu *et al.*, 2010). In epigenetic gene regulations, H3K27me3 and H3K4me3 are also considered as markers to define the repressive and active states of chromatin regions, respectively (Zhang *et al.*, 2007, 2009; Roudier *et al.*, 2011). To test whether *AS2* over-expression in the *iso-2D* mutant is also related to epigenetic regulations, a ChIP assay was first performed to analyse H3K27me3 and H3K4me3 levels, with two pairs of PCR primers corresponding to two separate regions in the *AS2* gene (Fig. 6A). Our result showed that the H3K27me3 level in the *AS2* gene was significantly reduced in the *iso-2D* but not in the *as2-5D* leaves (Fig. 6B). By contrast, the H3K4me3 level was significantly increased in *iso-2D*, but again not in the *as2-5D* leaves (Fig. 6C). These results indicate that molecular mechanisms in regulating *AS2* in the two mutants are different, and also suggest that the increased *AS2* expression level in *iso-2D* may involve epigenetic regulations.

## Discussion

**Fig. 7.**
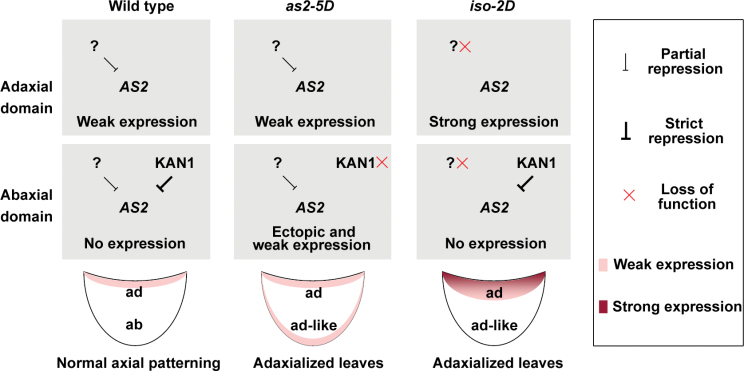
Model for the *AS2* expression patterns in the early stages of leaf development in Col-0, *as2-5D*, and *iso-2D*. The question mark indicates some proposed not-yet-known factors that are ubiquitously located in entire leaves to repress *AS2* expression. Action of the factors ensures *AS2* expression at a low level.

Because *AS2* plays a critical role in specifying leaf adaxial identity, regulation of the *AS2* gene must be important for leaf axial patterning. Based on previous knowledge and the results obtained in this study, models are proposed to explain the regulation of the *AS2* gene in *as2-5D* and *iso-2D* mutants during leaf polarity formation (Fig. 7). In wild-type leaves, *AS2* is expressed in the adaxial domain because of the abaxially located KAN1 proteins. In addition, the *AS2* expression level in wild-type leaves is very low, possibly due to the presence of some ubiquitously located not-yet-known factor(s) that represses *AS2* (Fig. 7, left column). Disruption of the KAN1-binding site in *as2-5D* leads to ectopic expression of *AS2* to the L1 layer on the abaxial leaf side, causing the *AS2*-over-expression phenotypes (Wu *et al.*, 2008). However, because the ubiquitously located factors function well in repressing *AS2*, expression of the *AS2* gene is still kept at a low level in L1 cells of both the adaxial and abaxial sides (Fig. 7, middle column). In *iso-2D*, however, the insertion of 35S enhancers blocks the function of the ubiquitously located factors, resulting in over-expression of *AS2*. Nevertheless, since the abaxially located KAN1 protein is still functional, the dramatically increased *AS2* expression is only limited in the adaxial leaf domain (Fig. 7, right column). In conclusion, it is proposed that, during leaf patterning, *AS2* is regulated both spatially and quantitatively in the entire leaf and both types of regulations are critical for the establishment of the leaf adaxial–abaxial polarity.

It was noticed that *FIL* repression occurs in both mutants, but to a greater extent in *iso-2D*. *AS2* expression driven by its native promoter is weakened after hte establishment of the leaf adaxial–abaxial polarity (see Supplementary Fig. S1 at *JXB* online), so that repression of *FIL* by *AS2* may also be weaker. Because *AS2* expression in the *as2-5D* mutant is driven by its native promoter, its expression level should be reduced along with leaf maturation. By contrast, the presence of 35S enhancers in the *iso-2D* allele ensures that *AS2* expression is maintained at high levels during all stages of leaf development which presumably explains the persistent repression of *FIL*. Our data showed that the leaf abaxially promoting genes *ARF3* and *YAB5* were also repressed in mature leaves of both *as2-5D* and *iso-2D* mutants, and *KAN1* and *KAN* were repressed in *iso-2D*. Compared with the *as2-5D* phenotypes, the *iso-2D* phenotypes are more severe. This is consistent with the analysed abaxially promoting genes that showed greater repression in *iso-2D* than in *as2-5D.* These results are also consistent with the previous suggestions: *ARF3* is a direct repressive target of the AS1–AS2 complex (Iwasaki *et al.*, 2013), and *AS2* and *KAN* genes may mutually repress each other’s transcription (Wu *et al.*, 2008).

Our *in situ* hybridization by analysing *AS2* expression showed that, in wild-type leaves, *AS2* transcripts are mainly concentrated in the L1 layer of the adaxial side. However, in *iso-2D* leaves, the *AS2* transcripts clearly form a gradient with the most concentrated part in the outmost cells. It is possible that the wild-type leaves may also possess the *AS2* transcript gradient, but the current techniques fail to detect it because of the very low level of *AS2* expression. The spatial and quantitative regulations of *AS2* together may facilitate the formation of a transcript gradient from the outmost adaxial epidermis to the inner cell layers. During organ patterning, formation of such a gradient of certain key regulatory factors could be a common mechanism. For example, the *Drosophila* Decapentaplegic morphogen gradient is essential for wing disc formation (Schwank and Basler, 2010). An additional example is the abaxially located miR165 and miR166 in *Arabidopsis*. Different from the gradient of *AS2* transcripts, the miR165 and miR166 gradients are present in the abaxial leaf domain with the highest level in the outmost epidermis (Yao *et al.*, 2009).

How *AS2* is quantitatively regulated is not yet known. Among many possible genetic pathways, epigenetic regulation could be one that plays roles in the quantitative regulation of *AS2*. Histone methylations have long been known to control gene expression and, in plants, several reports have provided evidence that histone methylations are involved in the quantitative regulation of gene expression (Jiang *et al.*, 2008; Schatlowski *et al.*, 2008; Liu *et al.*, 2010). More importantly, a recent study has demonstrated that a number of loci corresponding to the leaf polarity-controlling genes, including *AS2*, are modified by H3K27me3 (Lafos *et al.*, 2011). All these data suggest the possibility that epigenetic regulation may be involved in the control of the expression of leaf polarity genes. In this study, it is shown that the levels of the epigenetic markers H3K27me3 and H3K4me3 closely correlate with changes of *AS2* expression in the *iso-2D* leaves. Although this could be an explanation for the low level *AS2* expression in wild-type leaves, the possibility cannot be ruled out that the altered histone modification may simply be an indirect consequence of altered transcriptional regulation at the *AS2* locus. Thus, more detailed analysis is needed in the future to elucidate the molecular mechanism of the quantitative *AS2* regulations.

## Supplementary data

Supplementary data can be found at *JXB* online.

SupplementaryTable S1. List of primers used in this study.

Supplementary Fig. S1. *AS2* expression level is reduced in mature leaves. The qRT-PCR results were normalized to that produced by the primers at *ACTIN*, and the value of *AS2* in the mature leaves was arbitrarily fixed at 1.0. Bars show SE **, *P* <0.01.

Supplementary Data
