# Supplementary material for: Quantitative control of ASYMMETRIC LEAVES2 expression is critical for leaf axial patterning in Arabidopsis
Source: J Exp Bot. 2013 Sep 4;64(16):4895–905. doi: 10.1093/jxb/ert278 (PMC3830476; doi:10.1093/jxb/ert278)
Supplement: Supplementary Data [file supp_64_16_4895__index.html]

Quantitative control of ASYMMETRIC LEAVES2 expression is critical for leaf axial patterning in Arabidopsis — Quantitative control of ASYMMETRIC LEAVES2 expression is critical for leaf axial patterning in Arabidopsis — Supplementary Data 

# Quantitative control of *ASYMMETRIC LEAVES2* expression is critical for leaf axial patterning in *Arabidopsis*

## Supplementary Data

Data files

**Files in this Data Supplement:**

- Supplementary Data - Supplementary Data
