# Supplementary material for: Quantitative control of ASYMMETRIC LEAVES2 expression is critical for leaf axial patterning in Arabidopsis
Source: J Exp Bot. 2013 Sep 4;64(16):4895–905. doi: 10.1093/jxb/ert278 (PMC3830476; doi:10.1093/jxb/ert278)
Supplement: Supplementary Data [file supp_ert278_jexbot098020_file001.pdf]

Supplementary material

Quantitative control of *ASYMMETRIC LEAVES2* expression is critical for leaf axial patterning in *Arabidopsis*

Xiaofan Chen Hua Wang Jiqin Li Hai Huang Lin Xu[#]

National Laboratory of Plant Molecular Genetics, Shanghai Institute of Plant Physiology and Ecology, Shanghai Institutes for Biological Sciences, Chinese Academy of Sciences, 300 Fenglin Road, Shanghai 200032, China.

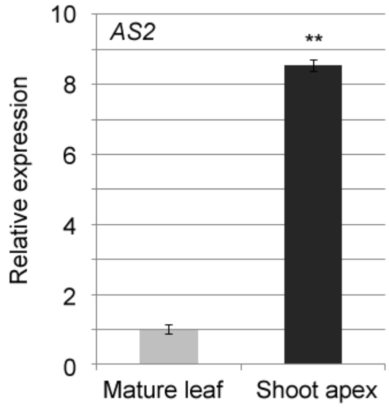

Fig. S1. *AS2* expression level is reduced in mature leaves. The qRT-PCR results were normalized to that produced by the primers at *ACTIN*, and the value of *AS2* in the mature leaves was arbitrarily fixed at 1.0. Bars show s.e. **, $P< 0.01$.

Table S1. List of primers used in this study.

Experiments	Primers	Sequence (5'→3')
ChIP		
	AS2-I-F	TCCATACGCATTCCAACTAC
	AS2-I-R	GGTAACTAGACCTAAGAGTC
	AS2-II-F	TTTTCAATGGCGGCTTTGTG
	AS2-II-R	CATTCCGGTTGACATTTTCG
	PI-II-F	CTGTCAAAGTCTCTCTTCAC
	PI-II-R	CTATCTCTTTCTCAATTTTAGG
	ACTIN-II-F	GGCTGAGGCTGATGATATTC
	ACTIN-II-R	CCATGATGTCTTGGCCTACC
qRT-PCR		
	AS2-F2	TCAGATAGATCTCAGCTGTG
	AS2-R2	AGCGATCGACGAAGACGAAC
	FIL-F2	GATTCCTAAAGCACCACCCG
	FIL-R2	GGACATGATAAACCCTAAG
	YAB5-F	GAAGCATTCAGCACTGCTGC
	YAB5-R	GTTACTCTCCAGCATTAGAC
	ARF3-F	CCAGAACTTTAGTACACGTA
	ARF3-R	ACTTTACTTAAGCCACAAGC
	KAN1-F	AACAAGCCTGCTGCTTCATC
	KAN1-R	ATCCAAATTGATCAAGAAAG
	KAN2-F	TCAGACGACTGGATGTTCG
	KAN2-R	ATCGAAACTAGTCAGTAGCC
	ACTIN-F	TGGCATCA(T/C)ACTTTCTACAA
	ACTIN-R	CCACCACT(G/A/T)AGCACAATGTT
Molecular cloning		
$AS2_{pro}$:GUS and *$mAS2_{pro}$:GUS*	$AS2_{pro}$-F	acgcgtcgacTGGTAGCTAGCGTTGTT GACAG
	$AS2_{pro}$-R	cgggatccTTTAATGACTTGAAAATGG AGTTTTTC
$35S_{pro}$:$AS2_{pro}$:GUS and *$35S_{pro}$:$mAS2_{pro}$:GUS*	$35S_{pro}$-F	gtcgacggatccAGATTAGCCTTTTCAAT TTCAG
	$35S_{pro}$-R	gtcgactctagaCGTGTTCTCTCCAAATG AAATG
In situ probe for *AS2*	AS2-in situ-F	AATTCCCTCTAGTACCAAATC
	AS2-in situ-R	ATCAATTAAGAGAGCAAGTCC

Note that lower case letters represent additional nucleotides to introduce restriction sites.
